# Zoonotic Bacteria in Fleas Parasitizing Common Voles, Northwestern Spain

**DOI:** 10.3201/eid2507.181646

**Published:** 2019-07

**Authors:** Ruth Rodríguez-Pastor, François Mougeot, Mª Dolors Vidal, Isabel Jado, Rosa M. González-Martín-Niño, Raquel Escudero, Juan José Luque-Larena

**Affiliations:** Universidad de Valladolid and Instituto Universitario de Investigación en Gestión Forestal Sostenible, Palencia, Spain (R. Rodríguez-Pastor, J.J. Luque-Larena);; Instituto de Investigación en Recursos Cinegéticos, Ciudad Real, Spain (F. Mougeot);; Universidad de Castilla-La Mancha, Ciudad Real (M.D. Vidal);; Instituto de Salud Carlos III, Madrid, Spain (I. Jado, R.M. González-Martín-Niño, R. Escudero)

**Keywords:** flea-borne diseases, rodent-borne diseases, vector, ectoparasites, transmission routes, small rodents, Microtus arvalis, vector-borne infections, bacteria, zoonoses, Spain, parasites, Francisella tularensis, Bartonella spp

## Abstract

We detected *Francisella tularensis* and *Bartonella* spp. in fleas parasitizing common voles (*Microtus arvalis*) from northwestern Spain; mean prevalence was 6.1% for *F. tularensis* and 51% for *Bartonella* spp. Contrasted vector–host associations in the prevalence of these bacteria suggest that fleas have distinct roles in the transmission cycle of each pathogen in nature.

A dynamic prevalence of *Francisella tularensis* and *Bartonella* spp. was reported in irruptive common vole (*Microtus arvalis*) populations during 2013–2015 from agricultural landscapes of northwestern Spain (*1*,*2*). In that area, notifiable tularemia has been endemic since 1997, and human cases periodically occur during outbreaks in voles (*3*,*4*). Prevalence of *F. tularensis* and *Bartonella* spp. in voles increases with vole density (*1**,**2*), highlighting the key role of fluctuating rodents in shaping zoonoses dynamics (*1**–**4*). Rodent ectoparasites often play a major role in transmitting zoonotic pathogens. In the population studied, ticks rarely infest voles (2% prevalence), whereas fleas are much more prevalent (68%) (*2*). Nevertheless, any potential role for vole fleas in the circulation of *F. tularensis* or *Bartonella* spp. in natural environments remains unknown. To elucidate realistic transmission route scenarios in host-dynamic environments (*5**–**8*), we investigated whether zoonotic bacteria occur concomitantly in voles and fleas.

Our main goal was to study the prevalence of *F. tularensis* in fleas collected from voles previously tested for tularemia (*1*). We screened flea DNA in search of 6 main zoonotic bacteria simultaneously (*Anaplasma phagocytophilum*, *Bartonella* spp., *Borrelia* spp., *Coxiella burnetii*, *F. tularensis*, and *Rickettsia* spp.), following the same molecular procedure (multiplex PCR) (*9*) previously used to screen vole pathogens (*1**,**2*). Voles and fleas were live-trapped in northwestern Spain during March 2013–March 2015 (Appendix). We collected fleas from each individual vole and identified and grouped them in pools (pool = total fleas/vole). Three flea species parasitize common voles in the area: *Ctenophthalmus apertus*, *Nosopsyllus fasciatus*, and *Leptopsylla taschenbergi* (*2*). We screened monospecific pools (all fleas in a pool belonged to the same species and came from the same vole host), for a sample size of 90 vole hosts (pools) and 191 fleas. We screened 78 *C. apertus* fleas (39 pools) and 113 *N. fasciatus* fleas (51 pools). Among the 90 voles providing fleas, 27 were *F. tularensis* PCR–positive; the remaining 63 were negative (*1*). Of these same 90 voles, 45 were *Bartonella* PCR–positive and 45 were negative. Seventeen were positive for both *F. tularensis* and *Bartonella* spp. (*2*).

Flea pools had an average of 2.12 fleas (range 1–9); however, most (>70%) contained 1 (51%) or 2 (22%) fleas (Table). We did not detect DNA from pathogens other than *F. tularensis* and *Bartonella* spp. in fleas. Three (3%) flea pools harbored *F. tularensis* DNA; we estimated the overall prevalence at 6%. *F. tularensis* prevalence in both flea species was low (1 positive pool of 51 in *N. fasciatus* and 2 of 39 in *C. apertus*). All *F. tularensis* PCR–positive flea pools came from *F. tularensis* PCR–positive voles, and prevalence of *F. tularensis* in fleas was significantly associated with its prevalence in voles (analysis of variance [ANOVA], R^2^ = 0.072, F_0.05, 1, 88_ = 6.81; p = 0.011). Of note, all fleas containing *F. tularensis* DNA were collected during July 2014, when vole populations reached top densities and tularemia prevalence peaked among them (33%) (*1*). The low prevalence of *F. tularensis* detected in fleas carried by infected hosts (3 of 27 pools) and the detection of infected flea pools only when abundance of the bacterium in the environment was highest (during vole peaks) (*1*,*4*) suggest that the quantitative role of fleas in the circulation of *F. tularensis* might be modest.

**Table:** Detection of *Francisella tularensis* and *Bartonella* spp. in 2 species of fleas from live common voles (*Microtus arvalis*), northwestern Spain, 2013–2015*

Voles	Flea species	Flea pools				Fleas		
		No.	*F. tularensis–*positive, %	*Bartonella* spp.–positive, %		No.	*F. tularensis* prevalence, % (range)	*Bartonella* spp. prevalence, % (range)
All	All	90	3.3	31.1		191	6.1 (3.3–8.8)	51.1([31.1–71.1)
	*Nosopsyllus fasciatus*	51	2.6	37.3		113	6.9 (3.9–9.8)	64.7 (37.3–92.2)
	*Ctenophthalmus apertus*	39	3.9	23.1		78	5.1 (2.6–7.7)	33.3 (23.1–43.6)
*F. tularensis*–negative	All	63				127	0	
	*N. fasciatus*	32				71	0	
	*C. apertus*	31				56	0	
*F. tularensis*–positive	All	27				64	20.4 (11.1–29.6)	
	*N. fasciatus*	19				42	18.4 (10.5–26.3)	
	*C. apertus*	8				22	25.0 (12.5–37.5)	
*Bartonella* spp.–negative	All	45				93		44.4 (26.7–62.2)
	*N. fasciatus*	21				53		71.4 (38.1–100)
	*C. apertus*	24				40		20.8 (16.7–25.0)
*Bartonella* spp.–positive	All	45				98		51.1 (31.1–71.1)
	*N. fasciatus*	30				60		60 (36.7–83.3)
	*C. apertus*	15				38		53.3 (33.3–73.3)

*Blank cells indicate that nothing can be calculated for that option.

Conversely, the role of fleas in the circulation of *Bartonella* spp. seems much more relevant. We detected *Bartonella* spp. in 28 (37%) flea pools and in both flea species (37% of *N. fasciatus* and 23% of *C. apertus*) (Table). We detected *Bartonella* spp. in fleas collected from *Bartonella* PCR–positive and *Bartonella* PCR–negative voles in nearly equal proportions (51% vs. 44%) (Table). The average prevalence of *Bartonella* spp. in fleas was not associated with its prevalence in voles (ANOVA, R^2^ = 0.006, F_0.05, 1, 88_ = 0.53; p = 0.467). We found a higher *Bartonella* spp. prevalence in *N. fasciatus* (65%) than in *C. apertus* (33%). We identified 3 *Bartonella* species among fleas (*B. taylorii* [17%], *B. grahamii* [14%], and *B. rochalimae* [3%]), as well as mixed infections (Appendix). These findings are in accordance with other research showing fleas as a main vector of *Bartonella* spp. (*5*). Although *F. tularensis* and *Bartonella* spp. have been simultaneously detected in ≈13% of voles during population density peaks (*2*), we identified no co-infection among flea pools (ANOVA, R^2^ = 0.011, F_0.05, 1, 88_ = 0.97; p = 0.328).

Our data show that *F. tularensis* and *Bartonella* spp. occur in the fleas infesting wild common voles in northwestern Spain, with notable differences in prevalence (6% and 51%, respectively) and associations with prevalence in vole hosts. Future studies are needed to determine the role of fleas in the circulation of these pathogens in nature and in particular to ascertain any effective vectoring of *F. tularensis*.

Appendix Aditional methods for a study of zoonotic bacteria in fleas parasitizing common voles, northwestern Spain.
