# Supplementary material for: Zoonotic Bacteria in Fleas Parasitizing Common Voles, Northwestern Spain
Source: Emerg Infect Dis. 2019 Jul;25(7):1423–5. doi: 10.3201/eid2507.181646 (PMC6590758; doi:10.3201/eid2507.181646)
Supplement: Appendix — Aditional methods for a study of zoonotic bacteria in fleas parasitizing common voles, northwestern Spain. [file 18-1646-Techapp-s1.pdf]

Article DOI: https://doi.org/10.3201/eid2507.181646

# Zoonotic Bacteria in Fleas Parasitizing Common Voles, Northwestern SpainReferences

## Appendix

### Study Area

The study was conducted in an intensified agricultural landscape in northwestern Spain. Fieldwork was conducted in 2 areas of 40 $km^2$ each in Palencia province, Castilla-y-León region (42°01′N, 4°42′W). The farmland of the study areas consists of a mosaic of crops dominated by non-irrigated cereals (≈48% of the agricultural surface), scattered with irrigated and non-irrigated alfalfa crops (≈10%) and other herbaceous crops. Natural and semi-natural habitats are reduced to small and dispersed patches of uncultivated land, pastures, or meadows (≈21% of the agricultural area) and a network of field margins (covering <5% of the agrarian surface) (for more details about the study area see [*1*]).

### Sample Collection

We held all the necessary licenses and permits for conducting this work: J.J.L.L., F.M., and R.R.P. held official animal experimentation licenses of level B-C for Spain, and capture permission (permit number 4801646) was provided by the Dirección General del Medio Natural, Junta de Castilla-y-León, Spain.

Common voles were live trapped in an agricultural area using LFAHD Sherman traps (8 cm × 9 cm × 23 cm) every 4 months (in March, July, and November) between March 2013 and March 2015 as is described in Rodríguez-Pastor et al. (*1*). Captured voles were taken to the lab alive, where they were euthanized through medical $CO_2$ inhalation, following a protocol approved by our institution ethics committee (CEEBA, Universidad de Valladolid; authorization code: 4801646). Immediately after death, the voles' fur was inspected carefully for fleas through

careful visual inspection and by gently blowing the vole's fur while holding the animal over a white plastic tray (520 × 420 × 95 mm) filled with water. We counted, collected and preserved in labeled tubes with 70% ethanol all the fleas collected from 225 individual voles. Each flea was later identified using a binocular microscope based on morphologic criteria following Gómez et al. (*2*). Although in a previous study, 240 common voles were screened for the occurrence of *Francisella tularensis* (*3*), for this current study, we used fleas collected from these same voles, but we only considered those animals that arrived alive to the lab to have a reliable estimation of flea infestation in the voles. Individual fleas often abandon carcasses of hosts that die in traps or during transport. From the 225 voles (141 voles were infested with fleas and 84 were not infested; Appendix Table 1), we selected voles that were infested with only 1 flea species, reducing the sample size to 90 individual voles (Appendix Table 1). Fleas from each vole were grouped in pools, and each pool was analyzed at molecular level (191 fleas in total, regrouped in 90 pools). A given pool consisted of fleas belonging to the same individual vole and flea species, i.e., 78 fleas in 39 pools were identified as *Ctenophthalmus apertus* (40.8%) and 113 in 51 pools as *Nosopsyllus fasciatus* (59.2%). Thus, the number of flea pools was equivalent to the number of sampled voles (n = 90) (Appendix Table 1). We did not analyze pools containing a mix of the 2 flea species, i.e., voles that simultaneously had fleas of both species were not considered in this study.

Flea pools were selected based on an a priori knowledge of *F. tularensis* prevalence in the voles that hosted them (*3*). In particular, from the initial 225 voles, 48 were *F.tularensis* PCR–positive and 177 were *F. tularensis* PCR–negative (Appendix Table 1). The proportion of voles infested with fleas and *F. tularensis* PCR–positive was 70.8% (34/48); while the proportion of voles infested with fleas and *F. tularensis* PCR–positive was ≈60.5% (107/177). We found that *F. tularensis* infection did not affect flea infestation. There were no significant difference between the proportions ($\chi^2$ 1.74, g.l. = 1, p>0.05). The selected 90 monospecific flea pools were made up of 27 flea pools from *F. tularensis* PCR–positive voles and 63 pools from *F. tularensis* PCR–negative voles. Since in a previous study we used a multiplex PCR method to analyze the DNA of the common voles that hosted the fleas studied here (see [*4*]; and PCR methods sub-section below), we also knew the prevalence of other zoonotic pathogens in these common voles, specifically *Anaplasma phagocytophilum*, *Bartonella* spp., *Borrelia* spp., *Coxiella burnetii*, *F. tularensis,* and *Rickettsia* spp.

## DNA Extraction

DNA from each flea pool was extracted using the QIAamp DNA Mini Kit (Qiagen, Hilden, Germany) according to the standard procedures of the manufacturer.

## PCR Methods

Pathogen detection in the DNA extracted from fleas was carried out using a multiplex PCR that simultaneously detected 6 vectorborne pathogens (*A. phagocytophilum*, *Bartonella* spp., *Borrelia* spp., *C. burnetii*, *F. tularensis*, and *Rickettsia* spp.), combined with a reverse line blotting (RLB), as previously described (*5,6*). Sensitivity of the multiplex PCR was between 10 and 100 GE (Genome Equivalents), and specificity with unrelated bacteria, mammals and arthropods was 100% (*5*). The same methodology was used to detect these same pathogens in common voles (*4*), including those hosting the fleas analyzed here. All the samples that tested positive to any given pathogen were further tested separately using specific probes with an individual PCR and subsequent RLB.

For detection of *F. tularensis* DNA in a flea pool, a phylogenetically informative region of *lpnA* (231 bp) was amplified by conventional PCR and further hybridization with specific probes by RLB, as previously described in Escudero et al. (*7*). Positive samples were tested for confirmation of the results using a real-time multitarget TaqMan PCR, targeting *tul4* and *ISFtu2* assays (*8*). A negative PCR control, as well as a negative control for DNA extraction, was included in each group of samples tested.

## Identification of *Bartonella* Species

*Bartonella*-positive samples were further analyzed using a multiplex PCR targeting the 16S rRNA and the intergenic transcribed spacer (ITS) 16S-23S rRNA. Subsequently, amplicons were analyzed with a RLB that included 36 probes for the identification of the different genotypes and species of *Bartonella* (*9,10*). Results are shown in Appendix Table 2.

## Statistical Analyses

As the number of fleas per pool ranged from 1 to 9, and all the fleas in each pool were screened together, we estimated an average pathogen prevalence per pool as the mean prevalence between the minimum and maximum prevalence. We assumed that either only 1 of the fleas was positive (minimum prevalence estimate) or that all the fleas from the pool were positive (maximum prevalence estimate). Average pathogen prevalence was estimated for all the fleas and for each flea species separately.

We used an analysis of variance (ANOVA) to test whether the pathogen prevalence in voles had an effect on the average pathogen prevalence in fleas. We also tested whether the average prevalence of a pathogen in fleas was related to the average prevalence of other pathogens in fleas. A $p<0.05$ was considered significant. Analyses were done with R v3.5.1 (*11*).

### References

1. Rodríguez-Pastor R, Luque-Larena JJ, Lambin X, Mougeot F. “Living on the edge”: the role of field margins for common vole (*Microtus arvalis*) populations in recently colonised Mediterranean farmland. Agric Ecosyst Environ. 2016;231:206–17. http://dx.doi.org/10.1016/j.agee.2016.06.041

2. Gómez López MS, Martín Mateo MP, Martínez MD. Malófagos, anopluros y sifonápteros. In: Barrientos JA, editor. Curso Práctico de Entomología. Asociación Española de Entomología, CIBIO: Centro Iberoamericano de Biodiversidad (Alicante), Universitat Autònoma de Barcelona; 2004. p. 599–614.

3. Rodríguez-Pastor R, Escudero R, Vidal D, Mougeot F, Arroyo B, Lambin X, et al. Density-dependent prevalence of *Francisella tularensis* in fluctuating vole populations, northwestern Spain. Emerg Infect Dis. 2017;23:1377–9. PubMed http://dx.doi.org/10.3201/eid2308.161194

4. Rodríguez-Pastor R, Escudero R, Lambin X, Vidal MD, Gil H, Jado I, et al. Zoonotic pathogens in fluctuating common vole (*Microtus arvalis*) populations: occurrence and dynamics. Parasitology. 2019;146:389–98. PubMed

5. Anda P, Escudero R, Rodriguez-Moreno I, Jado I, Jimenez-Alonso MI. Method and kit for the detection of bacterial species by means of DNA analysis. European patent EP1895015B1. 2012 July 25.

6. Jado I, Escudero R, Gil H, Jiménez-Alonso MI, Sousa R, García-Pérez AL, et al. Molecular method for identification of *Rickettsia* species in clinical and environmental samples. J Clin Microbiol. 2006;44:4572–6. PubMed http://dx.doi.org/10.1128/JCM.01227-06

7. Escudero R, Toledo A, Gil H, Kovácsová K, Rodríguez-Vargas M, Jado I, et al. Molecular method for discrimination between *Francisella tularensis* and *Francisella*-like endosymbionts. J Clin Microbiol. 2008;46:3139–43. PubMed http://dx.doi.org/10.1128/JCM.00275-08

8. Versage JL, Severin DDM, Chu MC, Petersen JM. Development of a multitarget real-time TaqMan PCR assay for enhanced detection of *Francisella tularensis* in complex specimens. J Clin Microbiol. 2003;41:5492–9. PubMed http://dx.doi.org/10.1128/JCM.41.12.5492-5499.2003

9. García-Esteban C, Gil H, Rodríguez-Vargas M, Gerrikagoitia X, Barandika J, Escudero R, et al. Molecular method for *Bartonella* species identification in clinical and environmental samples. J Clin Microbiol. 2008;46:776–9. PubMed http://dx.doi.org/10.1128/JCM.01720-07

10. Gil H, García-Esteban C, Barandika JF, Peig J, Toledo A, Escudero R, et al. Variability of *Bartonella* genotypes among small mammals in Spain. Appl Environ Microbiol. 2010;76:8062–70. PubMed http://dx.doi.org/10.1128/AEM.01963-10

11. R Core Team. R: A language and environment for statistical computing. R Foundation for Statistical Computing, Vienna, Austria. 2018 [2018 Apr 8]. https://www.R-project.org

**Appendix Table 1.** Distribution of common voles according to flea-infestation and *Francisella tularensis* PCR results in voles, northwestern Spain, 2013–2015

| Fleas in voles | *F. tularensis* PCR–positive voles | *F. tularensis* PCR–negative voles | Total voles | Observation |
|---|---|---|---|---|
| Only 1 flea species | 27 | 63 | 90 | Fleas used in this study |
| Mixed flea species | 7 | 44 | 51 | |
| Not infested voles | 14 | 70 | 84 | |
| Total | 48 | 177 | 225 | |

**Appendix Table 2.** Species-specific occurrence of *Bartonella* species in flea pools (n = 28), *Nosopsyllus fasciatus* pools, and *Ctenophthalmus apertus* pools according to infection type: single *Bartonella* species infection, or mixed-*Bartonella* species infection, northwestern Spain, 2013–2015

| *Bartonella* species | No. (%) | *N. fasciatus* (%) | *C. apertus* (%) |
|---|---|---|---|
| *B. grahamii* | 4 (14.3) | 4 (21.1) | 0 |
| With *B. rochalimae* | 2 (7.1) | 2 (10.5) | 0 |
| With *B. rochalimae* and *B. taylorii* | 7 (25) | 7 (36.8) | 0 |
| With *B. taylorii* | 6 (21.4) | 1 (5.3) | 5 (55.6) |
| With *B. elizabethae* | 3 (10.7) | 3 (15.8) | 0 |
| *B. taylorii* | 5 (17.9) | 1 (5.3) | 4 (44.4) |
| *B. rochalimae* | 1 (3.6) | 1 (5.3) | 0 |
| Total | 28 (100) | 19 (100) | 9 (100) |
